# Adiponectin, Leptin, IGF-1, and Tumor Necrosis Factor Alpha As Potential Serum Biomarkers for Non-Invasive Diagnosis of Colorectal Adenoma in African Americans

**DOI:** 10.3389/fendo.2018.00077

**Published:** 2018-03-12

**Authors:** Hassan Ashktorab, Akbar Soleimani, Alexandra Nichols, Komal Sodhi, Adeyinka O. Laiyemo, Gail Nunlee-Bland, Seyed Mehdi Nouraie, Hassan Brim

**Affiliations:** ^1^Department of Medicine, Cancer Center, College of Medicine, Washington, DC, United States; ^2^Islamic Azad University, Kashmar, Iran; ^3^Department of Surgery and Pharmacology, Translational Research, Marshall University Joan Edwards School of Medicine, Huntington, WV, United States; ^4^Endocrinology Division, College of Medicine, Howard University, Washington, DC, United States; ^5^University of Pittsburg, Medical Center, Pittsburg, PA, United States; ^6^Pathology Department, College of Medicine, Washington, DC, United States

**Keywords:** adiponectin, leptin, IGF-1, tumor necrosis factor alpha, biomarkers, colorectal adenomas, African Americans

## Abstract

The potential role of adiponectin, leptin, IGF-1, and tumor necrosis factor alpha (TNF-α) as biomarkers in colorectal adenoma is not clear. Therefore, we aimed to investigate the blood serum levels of these biomarkers in colorectal adenoma. The case–control study consisted of serum from 180 African American patients with colon adenoma (cases) and 198 healthy African Americans (controls) at Howard University Hospital. We used ELISA for adiponectin, leptin, IGF-1, and TNF-α detection and quantification. Statistical analysis was performed by *t*-test and multivariate logistic regression. The respective differences in median leptin, adiponectin, IGF-1, and TNF-α levels between control and case groups (13.9 vs. 16.4), (11.3 vs. 46.0), (4.5 vs. 12.9), and (71.4 vs. 130.8) were statistically significant (*P* < 0.05). In a multivariate model, the odds ratio for adiponectin, TNF-α, and IGF-1 were 2.0 (95% CI = 1.6–2.5; *P* < 0.001), 1.5 (95% CI = 1.5(1.1–2.0); *P* = 0.004), and 1.6 (95% CI = 1.3–2.0; *P* < 0.001), respectively. There was a positive correlation between serum adiponectin and IGF-1 concentrations with age (*r* = 0.17, *P* < 0.001 and *r* = 0.13, *P* = 0.009), TNF-α, IGF-1, and leptin concentration with body mass index (BMI) (*r* = 0.44, *P* < 0.001; *r* = 0.11, *P* = 0.03; and *r* = 0.48, *P* < 0.001), respectively. Also, there was a negative correlation between adiponectin and leptin concentrations with BMI (*r* = −0.40, *P* < 0.001), respectively. These data support the hypothesis that adiponectin, IGF-1, and TNF-α high levels correlate with higher risk of colon adenoma and can thus be used for colorectal adenomas risk assessment.

## Introduction

Colorectal cancer (CRC) is the third most commonly identified cancer in the United States for both genders (1). CRC in African Americans has a higher incidence and mortality in comparison to Whites. The mean age of CRC development in African Americans is lower than in Whites and Asian Pacific Islanders (2, 3). There is also evidence for a more proximal colonic distribution of lesions in African Americans (2). Epidemiologic studies have documented obesity as a risk factor for colorectal neoplastic transformation (4, 5). An association between body/abdominal fat and CRC was established (6). Fat tissue is progressively viewed not only as an energy storage site but also as an active endocrine tissue that produces and secrets proteins that act as hormones (7–9). So far, 20 adipohormones have been described. According to their physiological role, they have been divided into two groups: insulin resistance inducting factors such as resistin, tumor necrosis factor alpha (TNF-α), and interleukin 6 (IL-6) and insulin-sensitizing factors such as leptin, adiponectin, and visfatin (10, 11). Adipocytes’ secreted proteins might be the possible link between obesity and colon cancer. Indeed, adiponectin and leptin play an important role in energy homeostasis, glucose and lipid metabolism, immunity, bone formation, and regulation of body weight (12–14). These proteins can directly alter cancer risk by activating signal transduction pathways involved in carcinogenesis. They can also do so indirectly by acting on insulin sensitivity and the inflammatory response (15, 16).

Adiponectin is a unique adipokine, with two isoforms, secreted from abdominal fat tissue. It has antidiabetic, antiatherosclerotic, and anti-inflammatory effects (17) and directly modulates several intracellular signaling pathways involved in colorectal carcinogenesis (18–22).

Leptin, a 167 amino acid peptide, plays a vital role in the hypothalamus in relation to mammalian feeding behavior and energy expenditure and is a useful serum biomarker that reflects total body fat over a wide range of body mass indexes (BMIs) (23–25). Leptin was shown to promote colonic cell growth in *in vitro* and in human studies independently of insulin (26–28).

IGF-1, a growth and proliferation promotor, has also inhibitory effects on cell death and was reported as a major player in many disease processes including colon neoplastic transformation (29, 30).

One of the key chemical mediators implicated in inflammation-associated cancers is TNF-α which leads to activation of the NF-κB and AP-1 transcription factor complexes. TNF-α is frequently detected in biopsies from human cancer, produced by either epithelial tumor or stromal cells (31). TNF-α production by tumors has been associated with a poor prognosis, loss of hormone responsiveness, and cachexia/asthenia (32, 33).

The role of metabolic markers such as leptin, adiponectin, IGF-1, and TNF-α in the progression of diseases is becoming more significant. Indeed several studies have previously reported the correlation of such markers, individually, with colorectal neoplasia in different populations (34–38). However, none of these studies have addressed the association of these metabolic markers combined with the risk of colorectal neoplasia in a African Americans a population with higher prevalence of obesity, diabetes and colon cancer than other ethnic groups in the US population. Therefore, in the present study, we assessed the serum levels of these metabolites in an African American case–control study, where cases correspond to adenoma stages.

## Materials and Methods

### Study Population

Study participants were recruited from patients who underwent colonoscopy at Howard University Hospital either for screening or for diagnostic purposes between 2009 and 2015. The study was approved by the Howard University Institutional Review Board and consent forms were obtained from all participants. The total number of patients enrolled in this study was 378, including 180 cases (adenoma) and 198 controls (healthy African American controls with no colorectal lesions). Information about demographic characteristics, age, sex, weight, height, BMI, medical history, smoking, and other lifestyle exposures was collected from medical records. Within the control population, 14% presented with symptoms while only 10% of cases were symptomatic.

### Adiponectin, Leptin, TNF-α, and IGF-1 Analyses

The blood specimens were obtained from African American patients on the day of colonoscopy. Blood samples were centrifuged for 15 min at a speed of 3,500 *g* at 4°C then sera were separated and stored at −80°C. Serum concentrations of adiponectin (pg/ml), leptin (pg/ml), TNF-α (pg/ml), and IGF-1 (ng/ml), were quantified using commercially available ultrasensitive ELISA kits according to the manufacturer’s recommendations (Abcam, Cambridge, MA, USA).

### Statistical Analysis

Distribution of continuous data was presented by median (interquartile range) while categorical data was presented by tables of frequencies. Serum biomarkers were transformed to best normal distribution if required and were tested by *t*-test between cases and controls. Chi-square test was used for categorical data. A stepwise backward logistic regression analysis was applied to assess the significant relationship between biomarkers with risk of adenoma. Then we added important confounders in this model to assess the significant predictors of adenoma. We also used receiver operating characteristics curve (ROC) for each biomarker and test of different ROC to assess the diagnostic ability of different markers. All analyses were performed in STATA 14.0 (StataCorp., College Station, TX, USA). Two-tailed *P* < 0.05 were considered statistically significant.

## Results

### Characteristics of the Case and Control Groups

There were 111 (56%) and 91 (51%) females among controls and cases, respectively. The median ages and BMIs were 58 years and 28.5 for controls and 60 years and 28.4 for cases. There was no significant difference in age and gender between the two groups. The prevalence of overweight and obese subjects was significantly higher in the adenoma group (*P* = 0.026). Smoking was also higher in the case group (adenoma) [59 (33%)] vs. control group [36 (18%)], (*P* = 0.001) (Table 1). There were more diverticular lesions among cases than controls [109 (62%) vs. 96 (52%)] and hypertension [88 (49%) vs. 79 (40%)], respectively. The cases in this study had adenoma, primarily of the tubular type. No statistically significant differences were noted for all other parameters (Table 1). All controls’ sera were drawn from African Americans undergoing colonoscopy under the same condition as cases.

**Table 1:** Distribution of demographic, clinical, and inflammatory markers in case and controls.

	Controls		Cases		*P*-value^a^
			
	*N*	Results	*N*	Results	
Age, median (IQR)	198	58 (53–65)	180	60 (53–66)	0.2
Female gender	198	111 (56%)	180	91 (51%)	0.3
Body Mass Index, median (IQR)	198	28.5 (24.6–33.7)	180	28.4 (25.6–33.4)	0.8
Overweight and obese	198	140 (71%)	180	145 (81%)	0.026
Smoking	198	36 (18%)	180	59 (33%)	0.001
Alcohol	198	81 (41%)	179	78 (44%)	0.6
Exercise time per week, median (IQR)	198	3 (0–4)	180	3 (0–4)	>0.9
Location					
Ascending	–	–	180	154 (86%)	–
Transverse	–	–	180	8 (4%)	–
Descending	–	–	180	71 (39%)	–
Rectosigmoid	–	–	180	91 (51%)	–
History of colon disease	198	4 (2%)	180	1 (0.6%)	0.2
History of colon polyp	198	29 (15%)	180	29 (16%)	0.7
Family history of colon cancer	198	26 (13%)	180	32 (18%)	0.2
Reason for colonoscopy	196		180		0.3
Screening		117 (60%)		119 (66%)	
Follow-up		52 (26%)		44 (24%)	
Symptoms		27 (14%)		17 (10%)	
Diverticular disease	184	96 (52%)	177	109 (62%)	0.07
Dyslipidemia	198	38 (19%)	179	28 (16%)	0.4
Dyslipidemia medication	198	51 (26%)	180	44 (24%)	0.8
Diabetes	198	33 (17%)	179	32 (18%)	0.8
Diabetes medication	198	39 (20%)	179	37 (21%)	0.8
Hypertension	198	79 (40%)	179	88 (49%)	0.07
Hypertension medication	198	108 (55%)	179	109 (61%)	0.2
Aspirin	198	58 (29%)	180	60 (33%)	0.4

*^a^From t-test for continuous variables and chi-2 for categorical variables*.

### Metabolic Biomarkers in Adenoma Cases and Lesion-Free Controls

Serum concentrations of adiponectin (pg/ml), IGF-1 (ng/ml), TNF-α (pg/ml), and leptin (pg/ml) were significantly higher in adenoma group (Table 2). The respective differences in median adiponectin, IGF-1, and TNF-α levels between control and case groups [(11.3 vs. 46.0), (4.5 vs. 12.9), and (71.4 vs. 130.8), respectively] were statistically significant (*P* < 0.001). Median serum leptin concentration in adenoma cases was 16.4 vs. 13.9 in controls (*P* = 0.048).

**Table 2:** Serum adiponectin, IGF-1, tumor necrosis factor alpha (TNF-α), and leptin levels in cases and controls.

Biomarkers	Controls		Cases		
			
	*N*	Value	*N*	Value	*P*-value^a^
Adiponectin (pg/ml), median (IQR)	198	11.3 (1.5–26.9)	180	46.0 (21.4–115.2)	<0.001

IGF-1 (ng/ml), median (IQR)	198	4.5 (0.2–12.6)	180	12.9 (7.6–20.6)	<0.001

TNF-α (pg/ml), median (IQR)	198	71.4 (7.3–158.0)	180	130.8 (56.0–228.7)	<0.001

Leptin (pg/ml), median (IQR)	198	13.9 (4.5–36.5)	180	16.4 (3.1–28.7)	0.048

*^a^From t-test*.

### Correlation between Age, BMI and Analyzed Biomarkers

In controls, there was a positive correlation between serum adiponectin and IGF-1 concentrations and patients’ age. In both cases and control, there was statistically significant correlation between adiponectin, TNF-α, and leptin concentrations and BMI. Also, there was a positive correlation between IGF-1 concentration and BMI in cases (*r* = 0.25, *P* < 0.001) (Table 3).

**Table 3:** Correlation between age, Body Mass Index (BMI), and serum biomarkers, values are Pearson correlation (*P*-value).

	Age			BMI		
		
	All	Cases	Control	All	Cases	Control
Adiponectin^a^	0.17 (<0.001)	0.13 (0.07)	0.19 (0.007)	−0.40 (<0.001)	−0.44 (<0.001)	−0.41 (<0.001)
TNF^a^	0.06 (0.3)	−0.01 (0.9)	0.07 (0.3)	0.44 (<0.001)	0.70 (<0.001)	0.32 (<0.001)
IGF-1^a^	0.13 (0.009)	0.06 (0.4)	0.16 (0.03)	0.11 (0.030)	0.25 (<0.001)	0.05 (0.4)
Leptin	−0.01 (0.9)	−0.07 (0.4)	0.04 (0.6)	0.48 (<0.001)	0.60 (<0.001)	0.37 (<0.001)

*^a^Natural log*.

In a multivariate logistic regression analysis using the biomarkers, colon adenoma occurrence increased with increasing concentrations of adiponectin with an odds ratio (OR) of 2.0 (95% CI = 1.6–2.5; *P* < 0.001). Interestingly, this effect was limited to adiponectin of 100. The corresponding ORs for TNF-α and IGF-1 were 1.5 (95% CI = 1.5–2.0; *P* = 0.004) and 1.6 (95% CI = 1.3–2.0; *P* < 0.001) (Table 4). The OR for leptin was not significant in this analysis (*P* = 0.8). In this model, BMI could be replaced for TNF with OR: 1.06 (95% CI = 1.5(1.1–2.0)) for each unit increase. Smoking was also a significant predictor of higher risk of adenoma with an OR: 2.5 (95% CI = 1.4–4.5).

**Table 4:** Multivariate analysis of serum biomarkers in cases and controls.^a^

Biomarkers	Odds ratios (95% confidence intervals)	*P*-value
Adiponectin (natural log)^b^	2.0 (1.6–2.5)	<0.001
TNF (natural log)	1.5 (1.1–2.0)	0.004
IGF (natural log)	1.6 (1.3–2.0)	<0.001

*^a^Area under the curve (95% CI) for the model = 0.80 (0.75–0.84)*.

*^b^The effect of adiponectin was significant up to adiponectin of 100 (OR = 3.1 for each natural log). Then this effect was not significant (OR = 0.7, *P* = 0.3)*.

### Area under the Curve (AUC)

**Figure 1:**
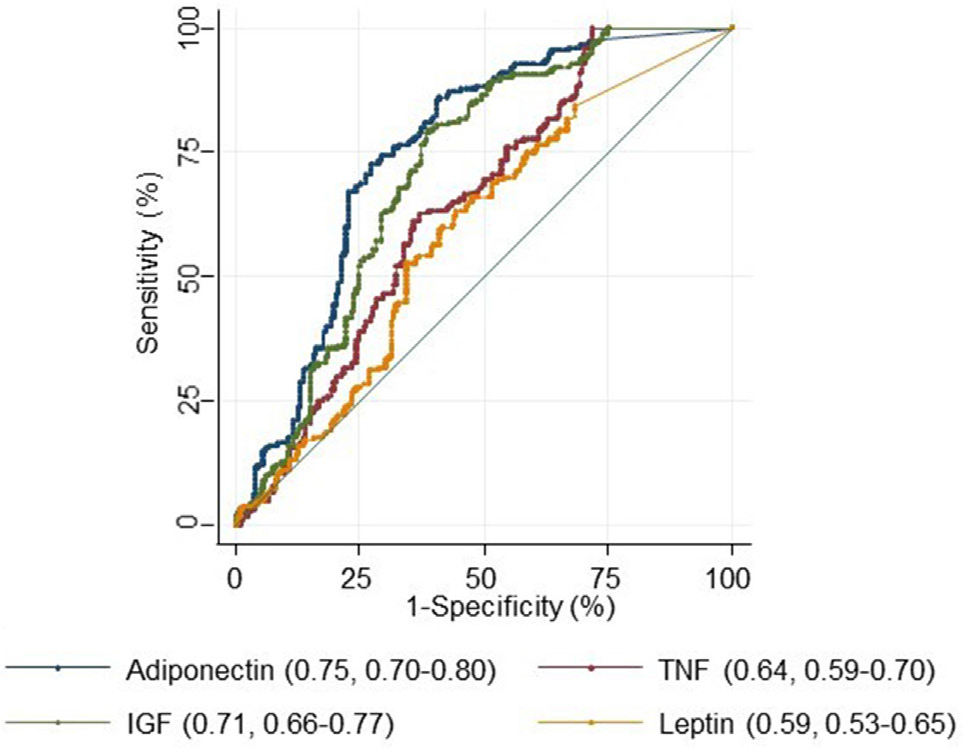
Receiver operating characteristics curve for adiponectin, tumor necrosis factor alpha (TNF-α), IGF-1, and leptin.

For each biomarker, the AUC with 95% confidence intervals was calculated to assess its usefulness as a sensitive and specific biomarker for colorectal adenoma diagnosis. The AUC for adiponectin, IGF-1, and TNF-α were 0.75 (0.70–0.80), 0.71 (0.66–0.77), and 0.64 (0.59–0.70). The AUC was lowest for leptin 0.59 (0.53–0.65) (Figure 1).

We then used the Youden index to select the optimum cut point for each serum marker to discriminate cases from controls. The best sensitivity was detected for an IGF > 6.84 and the best specificity and positive likelihood ratio were detected for an adiponectin >23.90 (Table 5).

**Table 5:** Discriminative power of serum biomarkers based on optimum cut point.

Biomarkers	Sensitivity	Specificity	Likelihood ratio+
Adiponectin > 23.90	73	73	2.67
IGF-1 > 6.84	79	61	2.04
TNF-α > 106.11	63	63	1.70
Leptin > 9.62	63	56	1.43

## Discussion

Colon adenoma is a precursor for colon cancer, if not detected early. Metabolic biomarkers are important indicators for prediction of colon disease. The present study explored serum adiponectin, leptin, IGF-1, and TNF-α association with colon adenoma. Our results showed significant differences in levels of serum adiponectin, IGF-1, and TNF-α in case (adenoma) and control groups (healthy normal). The OR for adiponectin, TNF-α, and IGF-1 were 2.0, 1.5, and 1.6, respectively. There was a positive correlation between serum adiponectin and IGF-1 concentrations with age, TNF-α, IGF-1, and leptin concentrations with BMI. However, there was a negative correlation between adiponectin concentration and BMI. Obesity has been reported to be a risk factor of colorectal adenoma and is known to be associated with CRC (39, 40). Obesity also occurs more frequently in African Americans than European Americans (41). Keimling et al. (42) reported increased colon cancer risks with higher BMI and waist circumference in men, but in women these anthropometric variables were unrelated to colon cancer. Obesity-related colorectal carcinogenesis is widely correlated with insulin resistance, which links colorectal adenoma to visceral obesity, physical inactivity and metabolic syndrome (43). As such, subtle non-invasive methods of assessing obesity-related molecules are of relevance in African Americans who suffer from obesity and CRC in a disproportionate manner. In the present study, we measured four serum biomarkers in patients with colon adenomas and compared their values to age- and gender-matched African American controls.

Adipocytokines have been the focus of intense study as novel risk biomarkers not only of metabolic syndrome but also of cancers (44). An inverse correlation between adiponectin level and colorectal adenoma has also been reported by several studies (20, 45, 46) which is not in agreement with our results. In addition, adiponectin levels were not associated with risk of colon adenoma in a Japanese case–control study (47) or in nested case–control studies of Norwegian and Swedish CRC patients (48, 49). In other case–control studies, the correlation between adiponectin level and CRC remains debatable (44, 50). However, we had no information regarding body weight changes in the patients and controls before the sampling, and consequently it was not possible to determine whether or not adiponectin levels’ increase was a result of obesity and adenoma outcome or a prior event. Our result is in line with Chong et al. and Inamura et al. who reported that high adiponectin plasma levels in colon cancer patients associate with poor prognosis and *KRAS* wild type tumors (51, 52). This study (51) showed among CRC patients, prediagnostic plasma adiponectin is associated with an increased risk of CRC-specific and overall mortality and is more apparent in patients with metastatic disease. They showed that adiponectin may be a marker for cancers that develop through specific pathways that may be associated with colorectal progression and poor prognosis.

Under high level of adiposity, macrophages are known to store in white adipose tissue, possibly in response to increasing levels of chemotactic signals. This leads to the secretion of a range of proinflammatory peptides from adipocytes and macrophages. In contrast to lean individuals, obese subjects’ adipose tissue expresses higher quantities of proinflammatory molecules such as TNF-α, IL-6, inducible nitric oxide synthase, and monocyte chemotactic protein-1 (53). Our result is consistent with the above studies reflecting an increase of leptin and TNF-α that is associated with BMI. Adiponectin suppresses the secretion of inflammatory cytokines such as TNF-α and induces the secretion of anti-inflammatory cytokines such as IL-10 (54). It has been reported to prevent tumor growth by suppressing angiogenesis *in vitro* and *in vivo* (55). Leptin is expected to be increased in obese patients such as our case population. In such patients, a decreased sensitivity to leptin occurs, resulting in failure to detect satiety, despite high energy stores. There is accumulating evidence that leptin signaling might be involved in colon cancer (56). Leptin activities are mediated through the transmembrane leptin receptor (ObR) (57), of which at least four isoforms of ObR with different COOH-terminal cytoplasmic domains have been described (58). The presence of ObRs and ObRl mRNA has been demonstrated in colon cancer cell lines, human colon tumors, polyps, and adjacent mucosa (56). It is observed that higher levels of leptin were linked with about a double increase in risk of CRC in men (59) which is consistent with our results. On the other hand, other studies show that no statistically significant differences were noted between patients with colorectal adenoma and normal control subjects (60, 61). In contrast, another study noted that serum leptin levels in patients with colon cancer were significantly decreased (7) despite lack of weight loss and BMI measurements when compared to control subjects.

Through a meta-analysis on IGF-1 and colorectal adenoma, Yoon et al. (62) reported that circulating levels of IGF-1, IGFBP1, and IGF-1/IGFBP-3 ratio were not associated with a risk of occurrence of colorectal adenoma but IGF-1 was associated with increased risk of occurrence of advanced colorectal adenoma. Our serum IGF-1 result is consistent with several studies (63, 64) that showed that higher IGF-1 level was significantly associated with increased risk of colorectal adenoma. However, most studies included in the meta-analysis (65) showed no such associations (63, 64). Kang et al. (66) did not observe any significant relationships between IGF-I and adenoma recurrence while another study resulted in IGF-I levels inverse association with colorectal adenoma recurrence and this inverse association was stronger for advanced adenoma recurrence than for non-advanced recurrence (67). These inconsistencies may be a result of different patient population, environmental impact, and methodology.

Recent evidence indicates that TNF-α serum level may be elevated in patients with colorectal adenoma that is consistent with our results (26, 68). However, our study is not in agreement with Vaughn et al. (69) that found no statistically significant associations between TNF-α and colon adenoma. Based on their investigation which focused on the associations between these cytokines and CRC (26, 70), it is possible these cytokines impact the progression of CRC rather than the initiation.

We established a positive correlation between serum adiponectin and IGF-1 concentrations and patients’ age. A study by Ryan et al. did not establish any age-related increase of adiponectin (71) with different BMI. However, this study did not include any colon adenoma. As a regulator of cell growth, IGF-1 was supposed to be equally, if not less, prevalent in the cases compared to control group. As such, its cell growth promoting features and increased serological quantity in cases is to be understood in the context of colonic neoplasia, rather than age itself.

There was also a positive correlation between TNF-α, IGF-1, and leptin concentrations and BMI. These findings are consistent with the status of these three markers in obese and overweight individuals. There was however a negative correlation between adiponectin concentration and BMI (*r* = −0.40, *P* ≤ 0.001 and Table 3). This finding is also expected as adiponectin is known to be inversely related to obesity and as such its increased concentration in our cases’ sera is to be assigned to colorectal neoplasia-associated processes. Indeed, in multivariate logistic regression analysis, colon adenoma risk increased with increasing concentrations of adiponectin with an OR of 2.0 (95% CI = 1.6–2.5; *P* ≤ 0.001). The corresponding ORs for TNF-α and IGF-1 were 1.5 (95% CI = 1.5–2.0; *P* = 0.004) and 1.6 (95% CI = 1.3–2.0; *P* ≤ 0.001) (Table 4). The OR for leptin was not significant in this model. Similarly, the AUC with 95% confidence intervals to assess the sensitivity and specificity of the biomarkers for colorectal adenoma diagnosis corresponded to 0.75 (0.70–0.80), 0.71 (0.66–0.77), and 0.64 (0.59–0.70) for adiponectin, IGF-1, and TNF-α, respectively. However, the AUC was lowest for Leptin. This study should be validated in a large sample size cohort. African-Americans have a higher obesity burden and are at high risk of colorectal adenoma and cancer. In conclusion, we demonstrated that circulating levels of adiponectin, TNF-α, and IGF-1 were associated with colorectal adenoma risk and as such might be good biomarkers for colorectal adenoma risk in African Americans.

## Ethics Statement

The study was approved by the Howard University Institutional Review Board (06-MED-39) and consent forms were obtained from all participants.

## Author Contributions

HA, HB, and SN study design and manuscript writing and critical evaluation of data; KS, AN, AL, GN-B, and AS collecting data/literature review; and SN performed statistical analysis.

## Conflict of Interest Statement

The authors declare that the research was conducted in the absence of any commercial or financial relationships that could be construed as a potential conflict of interest. The reviewer GM and handling Editor declared their shared affiliation.
